# Adhesion of gram‐negative rod‐shaped bacteria on 1D nano‐ripple glass pattern in weak magnetic fields

**DOI:** 10.1002/mbo3.640

**Published:** 2018-05-24

**Authors:** Iram Saleem, Samina Masood, Derek Smith, Wei‐Kan Chu

**Affiliations:** ^1^ Department of Physics and Texas Center for Superconductivity University of Houston Houston Texas; ^2^ Department of Physical and Applied Sciences University of Houston‐Clear Lake Houston Texas

**Keywords:** bacterial growth, cluster ion beam self‐assembly, *Escherichia coli*, nano‐ripple glass pattern, *Pseudomonas aeruginosa*

## Abstract

This research project has major applications in the healthcare and biomedical industries. Bacteria reside in human bodies and play an integral role in the mechanism of life. However, their excessive growth or the invasion of similar agents can be dangerous and may cause fatal or incurable diseases. On the other hand, increased exposure to electromagnetic radiation and its impact on health and safety is a common concern to medical science. Some nanostructure materials have interesting properties regarding facilitating or impeding cell growth. An understanding of these phenomena can be utilized to establish the optimum benefit of these structures in healthcare and medical research. We focus on the commonly found rod‐shaped, gram‐negative bacteria and their orientation and community development on the cellular level in the presence of weak magnetic fields on one dimensional nano‐ripple glass patterns to investigate the impact of nanostructures on the growth pattern of bacteria. The change in bacterial behavior on nanostructures and the impact of magnetic fields will open up new venues in the utilization of nanostructures. It is noticed that bacterial entrapment in nano‐grooves leads to the growth of larger colonies on the nanostructures, whereas magnetic fields reduce the size of colonies and suppress their growth.

## 1 INTRODUCTION

Bacteria is abundant in nature and may often incubate inside the human body. It is therefore important to understand its behavior in different environmental conditions, as well as the factors that influence the physiology of bacteria. One of the major known causes of illness is bacterial infection, which can prove fatal if proper treatment is not done. Microbial contamination is a severe issue in the healthcare and food industries, biomedical and clinical equipment, and water purification systems.

Bacteria plays its role in a variety of fields. It can be very harmful at times, and medical devices undergo bacterial colonization all the time. Because of this, scientists are trying to design antibacterial nanostructure materials. Many nanostructures are excellent impeders of bacterial growth and biofilms (Díaz, Cortizo, Schilardi, Gómez de Saravia, & Lorenzo de Mele, 2007; Epstein, Hochbaum, Kim, & Aizenberg, 2011; Hsu, Fang, Borca‐Tasciuc, Worobo, & Moraru, 2013; Lipovsky, Gedanken, & Lubart, 2013; Patil et al., 2015; Singh et al., 2016). Nanotechnology has many potential applications in the treatment and diagnosis of diseases and maintenance of medical equipment. Some engineered nanostructure polymer materials have been proven to inhibit the growth of bacteria, and thus act as antibacterial agents or coatings (Armentano et al., 2014). It has also been shown that nanostructured microspheres of silver zinc oxide resist the growth of bacteria and biofilms (Patil et al., 2014). The arrangement of bacterial colonies and formation of biofilms on nanopatterns facilitates metabolic reactions (Hochbaum & Aizenberg, 2010). Thus, it is essential to understand chemical and physical interactions between bacteria and nanosurfaces.

On the other hand, the nature of bacteria is very adaptive and how it survives under different conditions of density, temperature, pressure and magnetic field is of tremendous importance in medical and clinical science. Microorganisms have adapted to fluctuations in the Earth’s magnetic field over a period of time. However, the response of bacteria to other external magnetic field influences is still being investigated (Brkovic, Postic, & Ilic, 2015; Kohno, Yamazaki, Kimura, & Wada, 2000). In space, humans encounter a different environment of radiation and electromagnetic forces. One interest these days is to inspect the magnetic field influence on human bodies in outer space, outside of the influence of Earth’s gravity and its magnetic field. Electromagnetism has significant importance in understanding living organisms. Electromagnetic signals are received from the brain, heart, and isolated nerves, and signals are also emitted during the process of muscle building. Bacteria grow through the process of binary fission, and microbial growth strongly depends on factors such as ion transportation through the cell walls, their cellular structure, atomic and molecular interactions, enzymatic activities, other protein functions and the neural network in the living organisms. All these processes are facilitated by electromagnetic interactions. In some animals, bacteria consist of magnetic particles, mainly connected to the neural tissues, which determine the direction of their movement. The rapid increase in the use of magnetic field devices for medical use such as in radiology, cardiology, dentistry, oncology, and neurosurgery (Mornet, Vasseur, Grasseta, & Duguet, 2004) has made it important for us to understand the effect of magnetic fields on living organisms in the use of biomedical applications.

Strong magnetic fields can have strong effects (Hardell & Sage, 2008; Lai & Singh, 2004), such as the breaking and forming of chemical bonds which play a big role in the synthesis of DNA and thus cellular growth. However, weak magnetic field effects are not negligible. Although it may be challenging to design and conduct such experiments, they are of significant importance, as interaction with magnetic devices is unavoidable and continuous perturbation may result in permanent changes. Hence, the study of biological effects of magnetic fields is very crucial to understand microbial growth. The perturbative effects caused by weak magnetic fields have a huge impact on some organisms (Frankel, 1979; Masood, 2017). Since bacteria are single‐celled organisms, it is easy to study the effect of magnetic fields on their growth and cell structure, which may in turn be used to control and treat microbial diseases. This approach can later be extended to get detailed information about cellular structures.

The aim of this paper was to study the weak magnetic field influence on the growth of *E. coli* and *Pseudomonas aeruginosa* on nano‐ripple glass structures in comparison with plain glass cover slips. Some studies show that low frequency alternating magnetic fields tend to slow down the growth rate of *E. coli* (Aarholt, Flinn, & Smith, 1981; Cellini, 2008; Del Re, Bersani, Agostini, Mesirca, & Giorgi, 2004; Justo, Pérez, Alvarez, & Alegre, 2006; Segatore et al., 2012; Strasák, Vetterl, & Smarda, 2002). It is interesting to consider the adhesion of *E. coli* and its growth on nanostructure glass samples. The idea was influenced by the regeneration of the tissues and functional recovery following pathways set by nanostructures such as the spinal nerve cell growth guided by nanotubes or other biomaterial designs (Straley, 2010; Subramanian, Krishnan, & Sethuraman, 2009; Usmani et al., 2016). In this experiment, bacterial adhesion to the surface is enhanced due to the interaction of microorganisms with the nano‐ripple pattern. The bacterial colonies are bigger on nano‐ripple structures, but in the influence of weak magnetic fields, the colonies grow smaller in size. We studied the effect of both homogeneous (uniform electromagnetic field) and nonhomogeneous (nonuniform bar magnetic field) magnetic fields on the formation of bacterial colonies on nano‐ripple structures and plain glass cover slips.

## 2 EXPERIMENTAL SECTION

### 2.1 Fabrication of glass nano‐ripple

**Figure 1:**
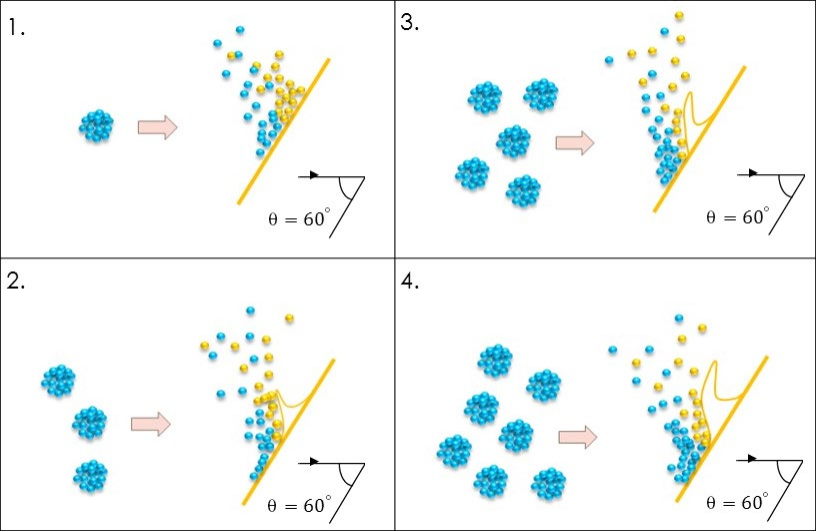
Schematic diagram of the oblique angle gas cluster ion beam (GCIB) irradiation used in the fabrication of nano‐ripple glass array structures

**Figure 2:**
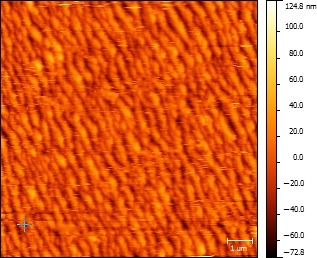
Atomic force microscope image of the nano‐ripple array on the glass surface obtained by GCIB irradiation fluence of 5 × 10^16^ clusters/cm^2^, for an incident angle of 60°

We engineer a nano‐ripple pattern on glass cover slips by means of oblique angle gas cluster ion beam (GCIB) irradiation (Kirkpatrick, 2003; Yamada, 1995; Yamada, Matsuob, Toyodaa, & Kirkpatrickc, 2001) (see Figure 1) using an Epion cluster ion implanter. Clusters of argon gas (each with about 3000 atoms of argon) of energy 30 keV bombard the glass surface at an incident angle of 60°. The off‐normal cluster ions and the surface atoms undergo synergistic interactions which cause the forward sputtering of atoms. As a result of forward sputtering and surface diffusion of the glass molecules, a nano‐ripple pattern is obtained (Lozano et al., 2013; Tilakaratne, 2012; Toyoda, Mashita, & Yamada, 2005). The geometry of the nano‐ripple structures is tunable by changing the GCIB irradiation fluence (Saleem et al., 2016). For the cases discussed, we irradiate the glass cover slip with a GCIB fluence of 5 × 10^16^ clusters/cm^2^. The cluster ion beam flux is kept constant at approximately 3.9 × 10^12^ clusters cm^−2^ sec^−1^. Cluster formation is a complicated process, and the theoretical descriptions of this process are limited. The phenomena of nucleation and growth take place through the birth of small molecular clusters that form and grow by molecular collisions. Cluster‐cluster aggregation becomes more evident when the number of the clusters are large. The detailed mechanism of GCIB induced ripple formation is explained in detail by Tilakaratne et al. in reference (Saleem & Chu, 2016). Figure 2 shows an atomic force microscope (AFM) image of the nano‐ripple arrays fabricated under selected conditions of 5 × 10^16^ clusters/cm^2^ GCIB fluence, and 60 degrees GCIB incident angle.

### 2.2 Experimental procedure

The glass substrates and the plates were thoroughly cleaned and autoclaved in order to remove all impurities and preexisting bacteria. Cultures of *Escherichia coli* and *Pseudomonas aeruginosa* were initially grown in liquid solutions, with each bacteria having a single mother culture. These mother cultures were vortexed, and then quickly pipetted in similar volumes onto multiple cleaned glass nano‐ripple structures, where they remained for 3 days at room temperature. We had three plates for each bacterial species growing on a plain glass for comparison. After sufficient growth, we washed the substrates with phosphate‐buffered saline (PBS) to remove the unattached bacteria from the surface. The bacteria was then stained using a gram crystal violet solution. The glass substrates were washed once again with the buffer solution to get rid of excess dye. The images of the bacteria on plain and nano‐ripple glass were taken under a microscope of 100× magnification.

**Figure 3:**
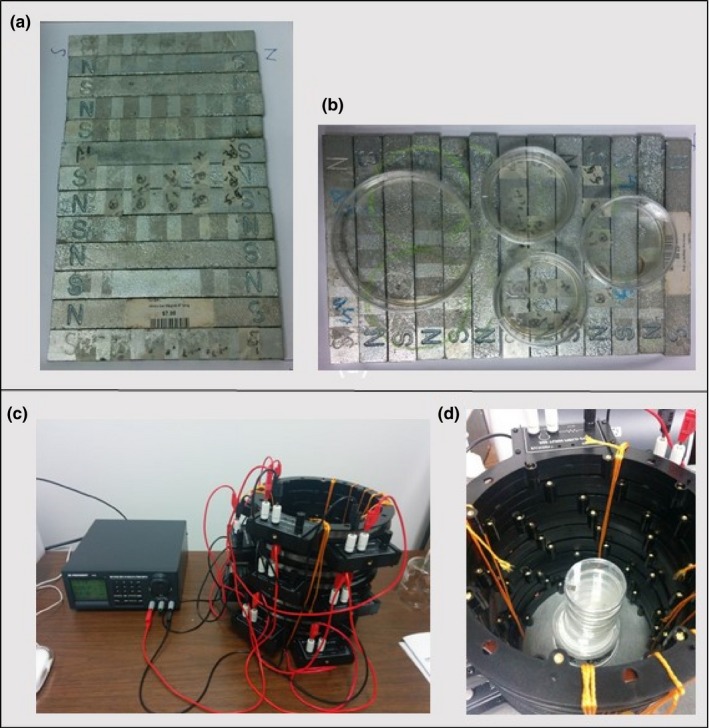
Experimental arrangement used for generating desired magnetic field environments. (a) and (b) show the bar magnetic field setup. (c) and (d) show the uniform magnetic field setup. The uniform magnetic field was generated by applying a constant current in the circular coils

**Figure 4:**
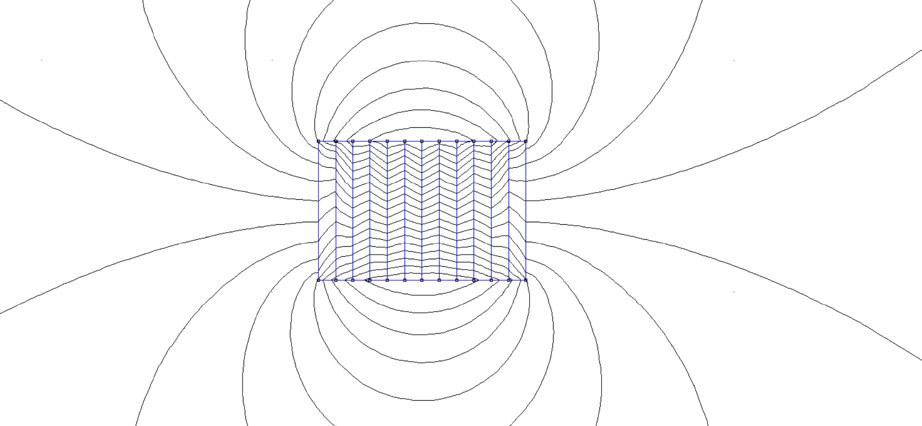
Representation of magnetic field lines from an alternating series of bar magnets. Figure generated using Finite Element Method Magnetics software (Muralidhar, Tree, & Dorfman, 2014)

Bacterial growth was studied at room temperature in two different types of weak magnetic fields. One set of *E. coli* plates was placed on a collection of permanent weak bar magnets arranged side‐by‐side as shown in Figure 3a,b, which generates a randomly distributed static weak magnetic field. This magnetic field varies rapidly within short distances, and cannot be represented in a well‐defined functional form. However, taking measurements at several nearby points, we noticed that the magnitude is always less than 5 Gauss (0.5 mT). A simple representation of the magnetic field lines due to these alternating magnets can be seen in Figure 4. The other arrangement was the uniform magnetic field generated by several interconnected Helmholtz coils with a weak DC current shown in Figure 3c,d. It is a weak static magnetic field of value 5 Gauss (0.5 mT). The inclusion of both uniform and nonuniform magnetic fields of similar maximum intensity allows us to check for effects which may be caused by the gradient of a magnetic field, rather than simply due to field intensity. For *Pseudomonas aeruginosa*, a set of plain and nanostructure glass surfaces were placed on the bar magnets. The experiment has been repeated more than three times with similar conditions and new cultures, and the reported results were reproduced.

## 3 RESULTS AND DISCUSSIONS

**Figure 5:**
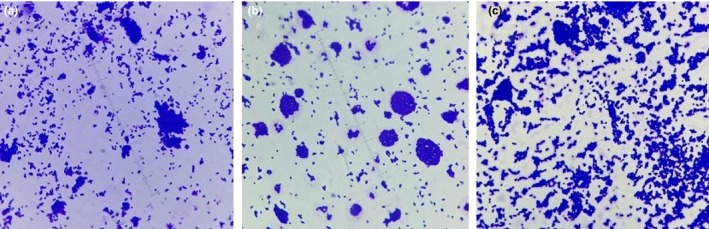
(a) and (b) show the growth of *Escherichia coli* on two nano‐ripple glass patterns in the absence of the magnetic field

**Figure 6:**
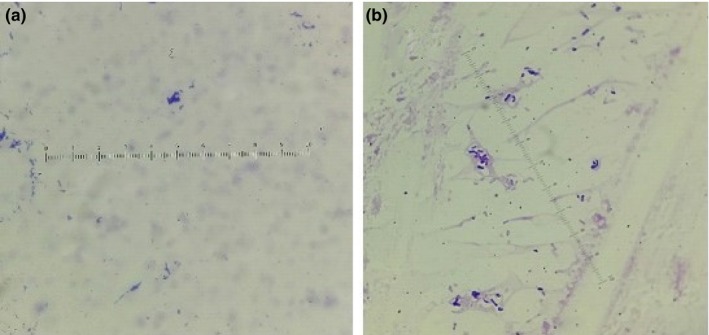
(a) and (b) show the growth of *Escherichia coli* on two plain glass surfaces in the absence of the magnetic field

It is noticed that *Escherichia coli* shows more growth on glass nano‐ripple surfaces than on plain glass. The nano‐ripple pattern traps the microorganisms in nano‐grooves of comparable size due to their alignment along the grooves and causes them to grow into larger colonies. Due to increased bacterial adhesion on the nanopattern, we observed an increase in the number of bacterial colonies compared to the ones on plain glass surfaces. Figure 5 shows microscopic images of *Escherichia coli* colonies on glass nano‐ripple structures in contrast to similar images on plain glass in Figure 6. The magnification scale is kept the same in Figures 5 and 6 for comparison. The difference in the sizes of colonies is significant on ripple structures (Figure 5) and the plain glass (Figure 6). The nano‐ripple structures behave as nanochannels for bacteria entrapment. Further investigation will help to develop tools to study the available theoretical models (Odijka, 2006) of cell confinement in nano‐grooves.

**Figure 7:**
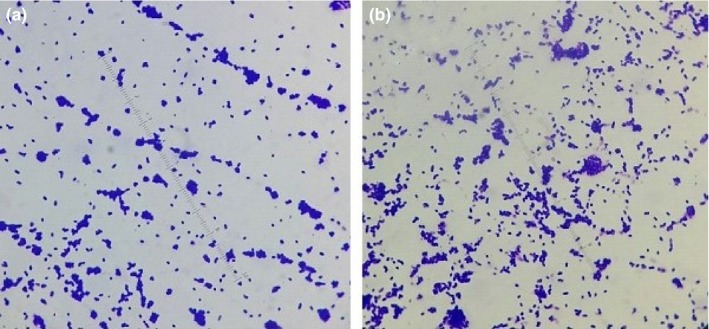
(a), (b) and (c) show the growth of *Escherichia coli* on three nano‐ripple glass substrates when grown over the bar magnets

**Figure 8:**
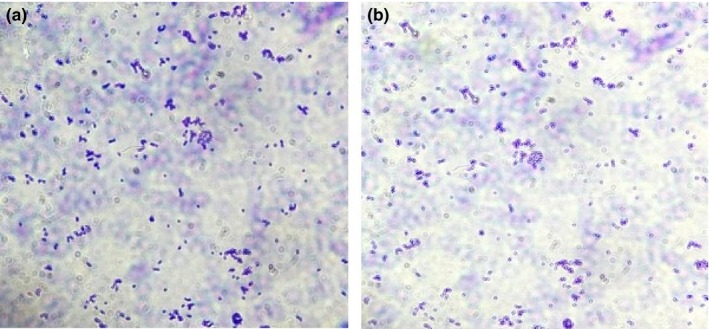
(a) and (b) show the growth of *Escherichia coli* on two nano‐ripple glass substrates in a uniform magnetic field

In addition, the structural effect was studied in the presence of magnetic fields. For this purpose, bacteria was simultaneously grown on nano‐ripple glass in uniform and nonuniform magnetic fields along with the plain glass. Figures 7 and 8 compare the bacterial growth on the bar magnets (nonuniform field) and in the uniform magnetic field and show that growth under the two magnetic fields affects the size of the colonies differently.

In the uniform field, the bacterial colonies spread themselves on the surface in a relatively uniform fashion and are small in size. In the nonuniform field, the colonies are relatively larger but still much smaller in comparison to the ones grown without the magnetic field’s influence, and the colonies are also not uniformly distributed over the surface.

A pattern in the distribution of bacterial colonies in Figure 7a,c is observed, which may be due to the alignment of bacteria along the magnetic field lines of bar magnets. Conversely, the random distribution of these colonies in Figure 7b could be due to the constructive and destructive interference of the fields from the multiple bar magnets, as the net effect of these magnetic fields varies from point to point and leads to an almost random distribution of field in certain regions over the magnets. However, further investigation of this effect is envisaged in future.

**Figure 9:**
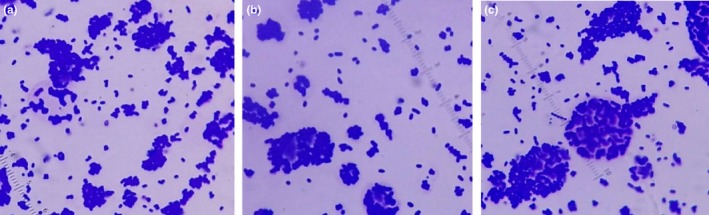
(a), (b), and (c) show the growth of *Pseudomonas aeruginosa* on two nano‐ripple substrates in the absence of magnetic field

**Figure 10:**
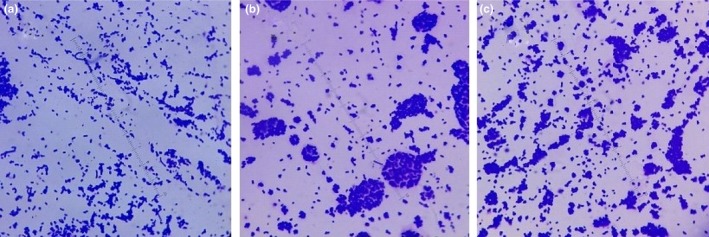
(a) and (b) show the growth of *Pseudomonas aeruginosa* on two nano‐ripple substrates when grown on the bar magnets

**Figure 11:**
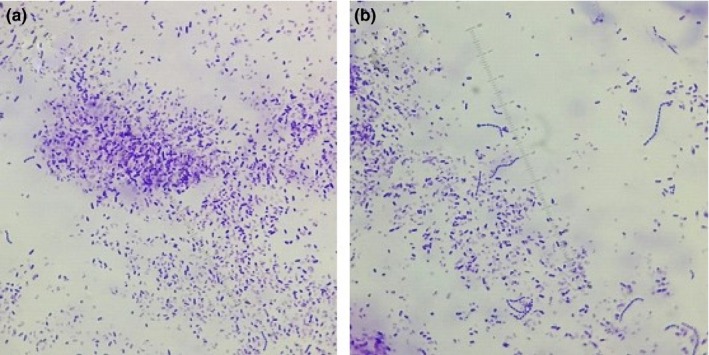
(a) and (b) show the growth of *Pseudomonas aeruginosa* on two plain glass surfaces in the absence of magnetic field

Magnetic field effects on the growth of *Pseudomonas aeruginosa* reveal similar results (Figure 9), but the colonies of *Pseudomonas aeruginosa* are larger in size than the *Escherichia coli*. This may be due to the difference in adhesive properties of the two bacterial species. In the influence of a magnetic field, bacterial colonies are smaller on glass nano‐ripple surfaces (Figure 10). As seen in Figure 11, on plain glass surfaces the adhesion of bacteria is comparatively less. Both pictures are taken with the same scale of magnification. There is more growth on nano‐ripple structures. This supports the idea that both rod‐shaped gram‐negative bacteria are similarly affected by the nano‐ripple pattern and the weak magnetic fields. However, due to the difference of size and adhesion properties of *Pseudomonas aeruginosa* and *Escherichia coli*, the bacterial colonies do not grow to equivalent sizes. The distribution pattern is also not significantly affected.

## 4 CONCLUSIONS

One‐dimensional nano‐ripple glass patterns formed by gas cluster ion beam irradiation show more adhesion of rod‐shaped bacteria as compared to plain glass slides. The growth of bacteria depends on the interaction of bacteria with nanostructures as well as the presence of a magnetic field. Uniform and nonuniform magnetic fields have different effects on the growth. Bacterial colonies are larger on nano‐ripple patterns than on the plain surfaces. Moreover, the magnetic field suppresses the bacterial growth on the structures. A comparison of bacteria growing on nano‐ripple glass patterns in different magnetic fields has shown different behavior as well. Uniform magnetic fields allow the colonies to distribute more uniformly on the nanostructure, but the colonies are smaller in size. It was also noticed that bacteria avoids adhesion to glass. Thus, around the glass slide we observe more growth. If the hypothesis of bacteria trapping in grooves is correct, it provides a rationale for an experimental test for the theoretical model of confined cells and their DNA in nanopatterns. If the effect on DNA is proved, it means that the behavior of bacteria will significantly change and their effect on the human body and the response to antibiotics or other drugs needs a detailed investigation. We have focused on the study of the rod‐shaped gram‐negative bacteria on a comparable size of nano‐grooves. However, these effects are expected to be different on different bacterial strains depending on the shape, size, and gram staining properties.

## ACKNOWLEDGMENTS

This work was partially funded by the state of Texas through the Texas Center for Superconductivity at the University of Houston. Partial support was provided by Faculty Research and Development Funds (FRSF) of University of Houston ‐ Clear Lake (UHCL). Authors would also like to acknowledge the support of the department of Biological Sciences and the chair Dr. Lawrence Rhode.

## CONFLICT OF INTEREST

There is no conflict of interest associated with the material of the paper.
